# Normal and cancer fibroblasts differentially regulate *TWIST1*, *TOX* and cytokine gene expression in cutaneous T-cell lymphoma

**DOI:** 10.1186/s12885-021-08142-7

**Published:** 2021-05-03

**Authors:** Syed Jafar Mehdi, Andrea Moerman-Herzog, Henry K. Wong

**Affiliations:** Department of Dermatology, University of Arkansas for Medical Sciences, 4301 West Markham St, #576, Little Rock, AR 72205 USA

**Keywords:** Cutaneous T-cell lymphoma, Mycosis fungoides, Tumor microenvironment, Fibroblasts, Biomarkers

## Abstract

**Background:**

Mycosis fungoides (MF) is a primary cutaneous T-cell lymphoma (CTCL) that transforms from mature, skin-homing T cells and progresses during the early stages in the skin. The role of the skin microenvironment in MF development is unclear, but recent findings in a variety of cancers have highlighted the role of stromal fibroblasts in promoting or inhibiting tumorigenesis. Stromal fibroblasts are an important part of the cutaneous tumor microenvironment (TME) in MF. Here we describe studies into the interaction of TME-fibroblasts and malignant T cells to gain insight into their role in CTCL.

**Methods:**

Skin from normal (*n* = 3) and MF patients (*n* = 3) were analyzed for FAPα by immunohistochemistry. MyLa is a CTCL cell line that retains expression of biomarkers *TWIST1* and *TOX* that are frequently detected in CTCL patients. MyLa cells were cultured in the presence or absence of normal or MF skin derived fibroblasts for 5 days, trypsinized to detached MyL

a cells, and gene expression analyzed by RT-PCR for MF biomarkers (*TWIST1* and *TOX*), Th1 markers (*IFNG*, *TBX21*), Th2 markers (*GATA3*, *IL16*), and proliferation marker (*MKI67*). Purified fibroblasts were assayed for *VIM* and *ACTA2* gene expression. Cellular senescence assay was performed to assess senescence.

**Results:**

MF skin fibroblast showed increased expression of FAP-α with increasing stage compared to normal. Normal fibroblasts co-cultured with MyLa cells suppressed expression of *TWIST1* (*p* < 0.0006)*,* and *TOX* (*p* < 0.03)*, GATA3* (*p* < 0.02) and *IL16* (*p* < 0.03), and increased expression of *IFNG* (*p* < 0.03) and *TBX21* (*p* < 0.03) in MyLa cells. In contrast, MyLa cells cultured with MF fibroblasts retained high expression of *TWIST1*, *TOX* and *GATA3*. MF fibroblasts co-culture with MyLa cells increased expression of *IL16* (*p* < 0.01) and *IL4* (*p* < 0.02), and suppressed *IFNG* and *TBX21* in MyLa cells. Furthermore, expression of *MKI67* in MyLa cells was suppressed by normal fibroblasts compared to MF fibroblasts.

**Conclusion:**

Skin fibroblasts represent important components of the TME in MF. In co-culture model, normal and MF fibroblasts have differential influence on T-cell phenotype in modulating expression of Th1 cytokine and CTCL biomarker genes to reveal distinct roles with implications in MF progression.

**Supplementary Information:**

The online version contains supplementary material available at 10.1186/s12885-021-08142-7.

## Background

Cutaneous T-cell lymphoma (CTCL) is a heterogeneous group of T cell malignancies that develop from the proliferation and transformation of mature skin-homing T cells, the most common types include mycosis fungoides (MF) and Sézary syndrome (SS) [1–3]. MF is an indolent variant that progressively advances primarily in the skin. Skin histology of early patch MF lesions show a low tumor burden with T cell infiltration characterized by Th1 cytokine bias, with increased expression of IL-2 and IFN-γ [3–6]. In addition, Th1 chemokines such as CXC chemokine ligand 9 (CXCL 9) and CXCL10 are also expressed in lesional skin of early CTCL, when epidermotropism of tumor cells is evident [7]. SS is an aggressive variant of CTCL characterized by erythroderma, lymphadenopathy and malignant T cells circulating in the blood. SS can have eosinophilia, a high level of IgE and chemokine ligand 17 (CCL17) in patients [8, 9]. MF and SS share similarities in gene expression and a subset of MF progresses to SS. Immune analysis of the skin in SS shows a Th2 cytokine profile [10] and the malignant T cells exhibit a Th2 cytokine pattern with increased IL-4 [11]. From gene profiling studies, a unique gene expression phenotype of SS has been uncovered [12]. Gene expression changes in SS, such as decreased expression of IFN-γ, and increased expression of unique biomarker genes identified in SS such as *TWIST1*, and *TOX* are frequent and represent important features of CTCL [13–15].

Recent studies have established that both the tumor microenvironment (TME) and the activity of tumor-infiltrating stromal cells affect cancer phenotypes [16]. The contribution of the TME to cancer prognosis was highlighted by a recent analysis of 39 malignancies that revealed that TME gene signatures are better predictors of survival than genes expressed in malignant tumor cells [17]. The TME is comprised of abundant fibroblasts and immune cells, as well as endothelial cells and extracellular matrix (ECM) components, which closely interact with tumor cells. Crosstalk between the TME and tumor cells can regulate cancer progression either positively or negatively. Fibroblasts have been shown to play an important role in maintaining the ECM and regulating epithelial differentiation by stromal–epithelial crosstalk to establish an invasion-permissive TME [18]. In B-cell lymphomas, fibroblasts have an inverse correlation with survival outcomes compared to carcinomas [19]. In CTCL, fibroblasts are an important component of the TME and have been shown to promote tumorigenesis by augmenting Th2 and attenuating Th1 immune responses [20]. In MF lesional skin, fibroblast-derived periostin promotes the production of thymic stromal lymphopoietin (TSLP) [21]. TSLP subsequently activates immature myeloid dendritic cells (DCs) to produce the Th2-attracting cytokine CCL17 [22], suggesting that fibroblasts from CTCL may nurture a Th2-dominant TME in MF lesions through TSLP secretion. Moreover, fibroblasts contribute to the high level of exotoxin-3 in lesional skin of CTCL, which interacts with CCR3 and controls the Th2-dominant TME in CTCL [23].

A Th1 bias has been described in the skin in early MF, when malignant cells are sparse, but how this immune bias develops is unclear. A Th1 cytokine pattern in the TME may suggest the presence of anti-tumor immunity that inhibits disease progression, which is consistent with an indolent clinical course of MF seen in the majority of patients. This observation was complemented by the finding that T cell clones isolated from early MF skin lesions lack a Th2-polarized cytokine pattern [24]. The interaction of tumor T cells with fibroblasts in MF is not well studied, but normal fibroblasts have variable activities in cancer and can exert suppressive functions against tumor cells [25]. With the indolent nature of MF, one hypothesis is that an interaction between skin fibroblasts and malignant T cells influences malignant T cell growth. The underlying propensity for immune bias is illustrated when culturing benign host T cells from SS patients in vitro away from the malignant Th2 cells, which leads to an enhanced Th1 cytokine pattern [26]. These findings suggest an important role for the TME in immune bias.

To better understand the interactions between fibroblasts and neoplastic T cells in CTCL, we studied immune changes and biomarker regulation using in vitro culture of skin fibroblasts and MF cells. Here we describe one of the first studies using a novel 2-dimensional co-culture method to demonstrate the immune regulation by skin fibroblasts of CTCL cells, and investigate how fibroblasts promote changes in CTCL.

## Methods

### Patient samples

Patient samples were obtained under approved institutional research protocol. Fibroblasts were isolated from lesional skin from MF patients (*n* = 3, stage IIB & IV, Table 1), and de-identified surgical skin remnants from age-matched healthy individuals (*n* = 3). Skin specimens were dissociated with 0.25% collagenase I (Worthington Biochemical, Lakewood, NJ) in explant medium (RPMI 1640 medium (Gibco, Gaithersburg, MD) supplemented with 20% fetal bovine serum (FBS, Atlanta Biologicals, Flowery Branch, GA) and 1% penicillin-streptomycin (Thermo-Fisher Scientific, Waltham, MA,) at room temperature for 1 h with agitation (Supplementary Fig. 1). Dissociated cells were filtered through a 40 μm cell strainer (Sigma Aldrich, St. Louis, MO), and then cultured for 2 to 4 days in explant medium, and further passaged to grow sufficient fibroblasts for co-culture experiment.

**Table 1:** Demographic details of patients and normal donors

SNO	Dx	Ethnicity	Approx Age	Sex	Stage	Characteristics
1	CTCL MF	Caucasian	47	F	IIb	Tumor
2	CTCL MF	Caucasian	78	F	IIb	Tumor
3	CTCL MF	Afro-American	54	M	IVA1	Plaques/Tumors
4	Normal Skin	Caucasian	66	M	Normal	
5	Normal Skin	Caucasian	66	F	Normal	
6	Normal Skin	Caucasian	50	F	Normal	

Staging has been done according to International Society for Cutaneous Lymphomas (ISCL)/European Organization for Research and Treatment of Cancer (EORTC) [27]

*Abbreviations*: *CTCL* Cutaneous T-cell lymphoma, *MF* Mycosis fungoides, *F* Female, *M* Male

### Cell lines

MyLa cells [28] were a kind gift provided by Dr. Michael Girardi (Yale University). HH and Hut78 cells were purchased from ATCC (Manassas, VA) in April 2019. Jurkat E6–1 clone was also purchased from ATCC (Manassas, VA). All the cell lines were first generation expansion of original stock. Jurkat, HH and MyLa cells were grown in RPMI 1640 medium (GIBCO, Gaithersburg, MD) supplemented with 10% FBS (Atlanta Biologicals, Flowery Branch, GA) and 1% penicillin-streptomycin (Thermo-Fisher Scientific, Waltham, MA). Hut78 cells were cultured in Iscove’s modified Dulbecco’s medium (IMDM) (GIBCO, Gaithersburg, MD) supplemented with 10% FBS and 1% penicillin-streptomycin. MyLa cells were confirmed to be negative for mycoplasma by universal mycoplasma detection kit (ATCC, Manassas, VA).

## 2-Dimensional co-culture model

**Fig. 1:**
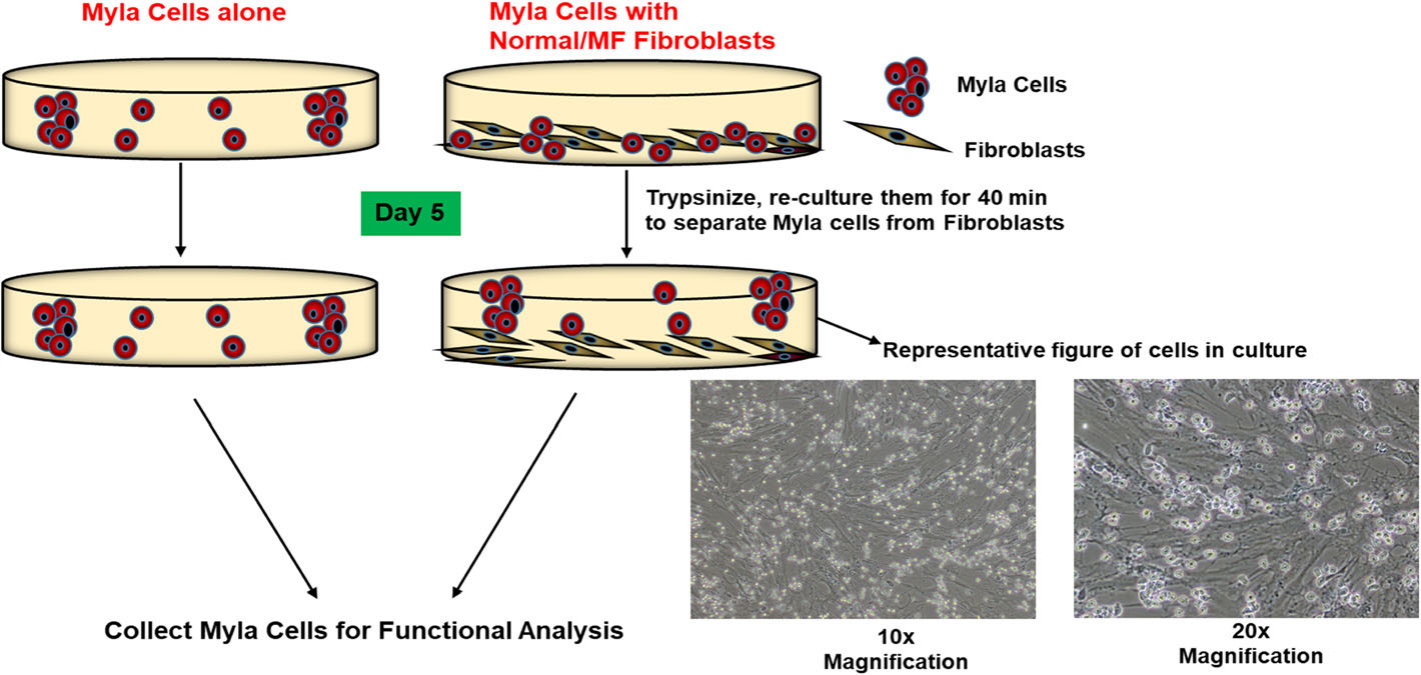
Schematic representation of 2-dimensional co-culture method

Primary fibroblasts were co-cultured with MyLa cells as previously described [29] (Fig. 1). Briefly, normal and tumor fibroblasts (5 × 10^5^ cells/ml) were seeded into separate 6-well plates, and cultured in RPMI 1640 medium containing 10% FBS and antibiotics until ~ 70% confluence was reached. Upon ~ 70% confluence, MyLa cells (3 × 10^5^/ml), derived from a patient with advanced MF, were added with fresh medium, and cultured with the fibroblasts for 5 days. MyLa cells were also cultured in the absence of fibroblasts as a control. After 5 days, co-cultured MyLa cells and normal/lesional fibroblasts were trypsinized and re-plated into new 6-well plates for 40 min to allow fibroblasts to adhere to the plastic, leaving MyLa cells in suspension. Separated MyLa cells along with their no-fibroblast controls were lysed for RNA extraction.

### RNA extraction and quantitative RT-PCR

RNA was purified using the RNeasy Plus mini kit, according to the manufacturer’s instructions (Qiagen, Hilden, Germany). cDNA was synthesized from 2 μg of total RNA using Maxima H Minus reverse transcriptase (Thermo-Fisher Scientific, Waltham, MA). Real-time PCR quantification was performed with Maxima SYBR Green qPCR master mix (Thermo-Fisher Scientific, Waltham, MA), on a QuantStudio 5 instrument (Applied Biosystems, Foster City, CA).

### Immunohistochemistry

Immunohistochemistry (IHC) staining was performed on paraffin sections with anti-FAPα (ab207178, 1:200; abcam, Cambridge, MA). After performing heat mediated antigen retrieval with Tris/EDTA buffer (pH 9.0) for 30 min, slides were blocked with normal blocking serum for 20 min, followed by incubation with anti-FAPα antibody for 2 h after serial washing for 5 min. Assays were completed with Vectastain elite ABC peroxidase (HRP) kit (Vector laboratories, Burlington, ON) and counterstaining with haematoxylin. A Zeiss AXIO Imager.M2 microscope (Zeiss, Nashville, TN) was used to obtain images at 20x and 40x magnification with an Olympus DP73 digital camera (Olympus, Melville, NY) with 14-bit bit depth and 17.28mb pixel resolution on bright field mode. Images were taken using CellSens entry 1.17 software (Olympus, Melville, NY) at 72 dpi horizontal and vertical resolution with 2400 × 1800 dimensions. Images were further processed to 600 dpi horizontal and vertical resolution with 8000 × 4500 dimensions using Photoshop CS6 (64 bit) (Adobe, San Jose, CA).

### Cellular senescence

Cellular Senescence was assessed with the cellular senescence assay kit (Cell BioLabs, San Diego, CA) according to the manufacturer’s protocol that detects senescence-associated β-galactosidase (SA-βGal) in cells.

### Statistical analysis

Two-tailed Student’s *t*-test was used to analyze the quantitative RT-PCR data for mRNA expression, with *P* < 0.05 considered statistically significant. All data is the mean of three separate experiments, and results are presented as mean ± standard deviation.

## Results

### MF lesional fibroblasts are phenotypically different from healthy skin fibroblasts

**Fig. 2:**
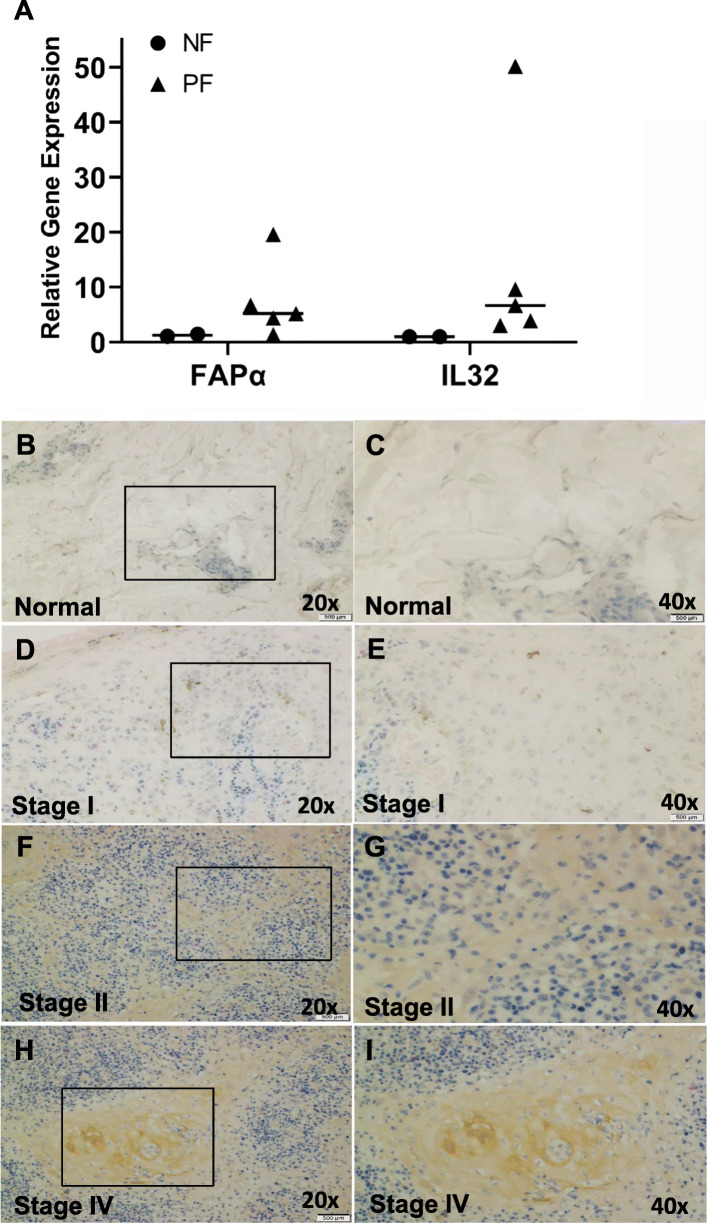
Expression of *IL32* and *FAPα* in normal and MF fibroblasts. **a** *IL32* and *FAPα* gene expression in normal and MF fibroblasts by RT-PCR (NF: Normal Fibroblast; PF: Patient Fibroblast). **b**-**e** Identification of in situ and primary culture FAP-α+ CAFs in normal (*n* = 3) and MF (*n* = 3). Representative IHC images of FAP-α staining showing a strong positive CAFs in MF stage IV tissue than normal skin. Stage II (**f**, **g**) shows low to moderate FAP-α positive CAFs compared to stage I (**d**, **e**) and normal (**b**, **c**). Scale bars, 500 μm

Due to previously observed effects of MF lesional skin fibroblasts on T cell polarization, we hypothesized that there are inherent phenotypic differences between fibroblasts from MF lesional skin and healthy skin. Therefore, we compared gene expression for cancer-associated fibroblast (CAF) markers including *MMP2* [30], *MMP9* [31], *MMP21* [32], *TGFA* [33], *CXCL12* [34], *ITGA3* [35], *FAPα* [34], and *IL32* [36] in fibroblasts derived from normal skin and MF tumors. Of the genes analyzed, only *IL32* and *FAPα* demonstrated differential expression between MF and normal fibroblasts (Fig. 2a). For FAP-α, we further compared expression by immunostaining tissue sections from normal skin and MF lesional skin. FAP-α expression was low in normal skin (Fig. 2b-c), but higher in MF lesional skin (Fig. 2d-i). Furthermore, FAP-α staining was more intense in MF stage IV lesional skin (Fig. 2h-i) compared to lesional skin from stages I-II (Fig. 2d-g). The staining of FAP-α appears to increase with stage. Thus, normal and MF fibroblasts are phenotypically different, but do not share many CAF markers with other solid tumors.

### Short-term co-culture does not affect expression of fibroblast markers

**Fig. 3:**
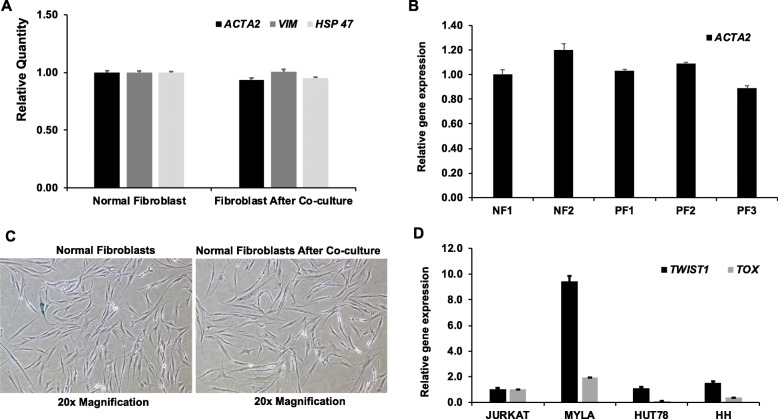
**a** Expression of fibroblast markers: Fibroblasts separated after co-culture were analyzed for gene expression by RT-PCR to quantify changes in the expression of known fibroblast markers (*VIM*, *HSP47*) and a gene which is expressed by both fibroblasts and cancer-associated fibroblasts (*ACTA2*). **b** *ACTA2* gene expression in normal and MF fibroblasts by RT-PCR (NF: Normal Fibroblast; PF: Patient Fibroblast). **c** Cellular Senescence: A colorimetric based cellular senescence assay based on quantification of the senescence-associated beta-galactosidase (SA-β-gal or SABG) was performed. Co-culture of fibroblasts with MyLa cells had no effect on fibroblast proliferation. **d** MF biomarker expression in cell lines: MyLa, HH and Hut78 cell lines were tested for MF biomarkers (*TWIST1* and *TOX*). Jurkat cells were used as a control. Only MyLa cells showed expression of *TWIST1* and *TOX* compared to Jurkat cells

The histologic findings suggest a role of fibroblasts interacting with malignant cells in CTCL. To study interactions between fibroblasts and malignant cells in CTCL, we determined if short-term co-culture of fibroblasts with MyLa cells altered the phenotype of normal fibroblasts. We assessed for changes in fibroblast markers, mesenchyme-specific genes such as vimentin (*VIM*), alpha-smooth muscle actin (*ACTA2*) and heat-shock protein 47 (*HSP47*) by quantitative gene expression analysis. After co-culture with MyLa cells, these genes were observed to be unchanged in normal fibroblasts (Fig. 3a). *ACTA2* is a marker for CAFs in solid tumors [37], and is associated with worse clinical outcome for several cancers including breast and lung cancers [38, 39]. In contrast to CAFs from pancreatic and colorectal carcinomas, where expression of *ACTA2* is elevated [40, 41], *ACTA2* expression was very similar in MF and normal fibroblasts after short-term co-culture with MyLa cells (Fig. 3b).

In addition, we also measured cellular senescence by quantifying SA-βGal activity, and found that co-culture with MyLa cells did not induce a detectable senescence phenotype in normal fibroblasts (Fig. 3c). Therefore, short-term co-culture of MyLa cells with normal fibroblasts does not induce any change in fibroblasts in terms of phenotypic marker expression and proliferation capacity.

### Normal fibroblasts alter expression of CTCL biomarkers in CTCL cells

Expression of *TWIST1* and *TOX* is frequently increased in tumor T cells from CTCL patients [42, 43]. Therefore, we assessed the CTCL cell lines MyLa, Hut78 and HH for expression of *TWIST1* and *TOX*. Of the three cell lines analyzed, only MyLa cells expressed these CTCL biomarker genes (Fig. 3d), indicating that abnormal gene expression similar to that seen in patient-derived T cells is preserved MyLa cells.

**Fig. 4:**
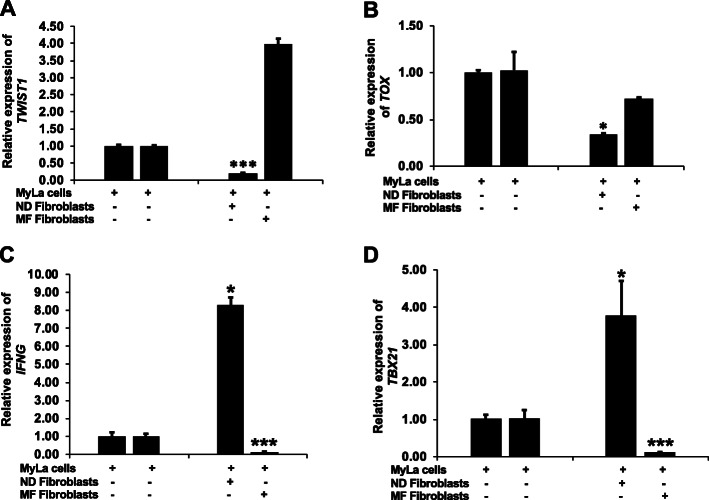
Normal fibroblasts suppresses MF biomarker expression in MyLa cells: **a**, **b** Expression of MF biomarker genes (*TWIST1* and *TOX*) are significantly suppressed in MyLa cells after co-culture with normal fibroblast (*TWIST1*: *p* < 0.0006; *TOX*: *p* < 0.03) as measured by RT-PCR. Co-culture of MyLa cells with MF fibroblasts does not show effect on MF biomarkers gene expression. Data represented here is the mean of three (*n* = 3) different experiment and paired *t-test* has been performed to calculate the *p* value. **c**, **d** Normal fibroblasts promotes Th1 environment in MyLa cells: Expression of Th1 cytokine and marker genes (*IFNG* and *TBX21*) are significantly increased in MyLa cells after co-culture with normal fibroblast (*IFNG*: *p* < 0.03; *TBX21*: *p* < 0.03), whereas co-culture of MyLa cells with MF fibroblasts further suppresses Th1 cytokine and biomarker gene expression in MyLa cells

To study the influence of the TME in CTCL, MyLa cells were co-cultured with normal fibroblasts (*n* = 3) or MF lesional fibroblasts (*n* = 3), and changes in the expression of CTCL biomarker genes in MyLa cells were assessed. As shown in Fig. 3d, MyLa cells have endogenously high *TWIST1* expression, but after co-culture with normal fibroblasts, expression of *TWIST1* was significantly reduced (*p* < 0.0006) (Fig. 4a). *TOX* expression was also suppressed in MyLa cells after co-culture with normal fibroblasts (*p* < 0.03, Fig. 4b).

In contrast, MyLa cells co-cultured with MF lesional fibroblasts retained high expression of *TWIST1*, and expression of *TWIST1* tended to increase (Fig. 4a). TOX remained higher when co-cultured with MF fibroblasts (Fig. 4b). As TOX plays an important role in CTCL proliferation [44] and T cell exhaustion [45], the ability of normal fibroblasts to suppress *TOX* expression in MyLa cells suggests that fibroblasts in the MF TME may have a role in regulating T cell exhaustion and disease progression.

### Normal fibroblasts promote a Th1 phenotype in CTCL cells

TWIST1 has been shown to limit the expression of *IFNG* and *TBX21* in Th1 cells [46]. Therefore, we examined the effect of the co-culture model on the expression of the *IFNG* and *TBX21* gene in MyLa cells. Co-culture with normal fibroblasts increased the expression of both *IFNG* (*p* < 0.03) and *TBX21* (*p* < 0.03, Fig. 4c-d). *TBX21* encodes T-box transcription factor (T-bet), a master-regulator of Th1 differentiation [47]. Given the modulatory role of TWIST1 in Th1 differentiation [46], the increased expression of *IFNG* and *TBX21* may be secondary to suppression of *TWIST1* in MyLa cells co-cultured with normal fibroblasts. In contrast, co-culturing MyLa cells with MF tumor-derived fibroblasts significantly suppresses the expression of *IFNG* (*p* < 0.0002) and *TBX21* (*p* < 0.0004) in MyLa cells (Fig. 4c-d). These findings suggest that normal fibroblasts promote a Th1-like transcriptional network in MyLa cells.

### Normal fibroblasts attenuate Th2-bias and proliferation in the TME

**Fig. 5:**
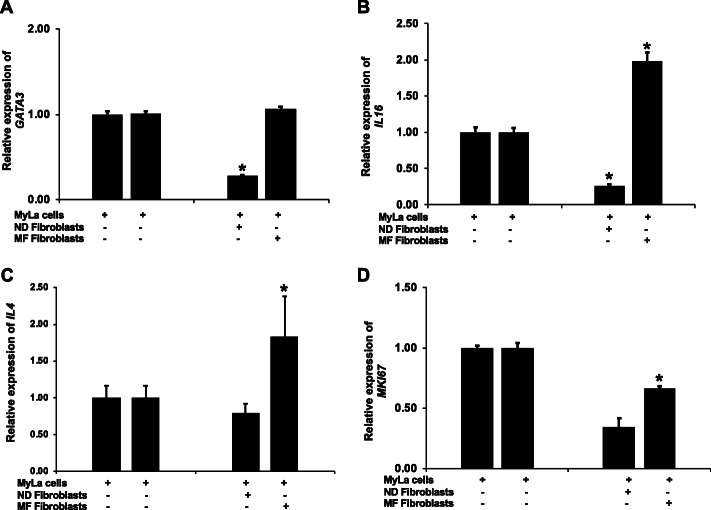
**a** Normal fibroblasts attenuates Th2 processes in MyLa cells: Expression of *GATA3* is significantly suppressed in MyLa cells after co-culture with normal fibroblasts (*GATA3*: *p* < 0.02) as measured by RT-PCR, whereas co-culture of MyLa cells with MF fibroblasts does not show effect on *GATA3* expression. Data represented here is the mean of three different experiment and paired *t-test* has been performed to calculate the *p* value. **b**, **c** Normal fibroblasts regulates cell growth in MyLa cells: **b** Expression of *IL16* is significantly suppressed in MyLa cells after co-culture with normal fibroblast (*p <* 0.03) in a short term culture, whereas co-culture of MyLa cells with MF fibroblasts increases the expression of *IL16* expression in a significant manner (*p* < 0.01) as measured by RT-PCR. **c** Expression of *IL4* was suppressed in MyLa cells after co-culture with normal fibroblasts in a short term culture, whereas co-culture of MyLa cells with MF fibroblasts significantly increases the expression of *IL4* expression (*p* < 0.02) as measured by RT-PCR. **d** Co-culture of MyLa cells with normal fibroblasts further suppresses *MKI67* expression in MyLa cells whereas co-culture with MF fibroblasts does not show noticeable effect on *MKI67* expression. Data represented here is the mean of three different experiments and paired *t-test* has been performed to calculate the *p* value

Studies have shown that T-bet not only promotes Th1 differentiation, but also represses Th2 differentiation by suppressing *GATA3* expression [48] and reducing the binding of GATA3 to DNA [46]. GATA3 is crucial for the differentiation of naïve CD4+ T cells into Th2 cells. Furthermore, *GATA3* deletion permits the development of IFN-γ producing cells [49]. Therefore, we analyzed whether *GATA3* expression in MyLa cells is affected by co-culture with fibroblasts. After co-culture with normal fibroblasts, *GATA3* expression was suppressed in MyLa cells (*p* < 0.02, Fig. 5a). In MF, *GATA3* expression is increased and high expression of *GATA3* was retained in MyLa cells after co-culture with MF tumor-derived fibroblasts (Fig. 5a).

Upregulation of IL-16 in advanced CTCL can augment the growth of malignant T cells in an autocrine manner [50]. IL-16 is a potent T-cell chemoattractant and a known marker of MF onset and stage [50]. Based on the role of IL-16 as a regulator of T-cell proliferation and migration, we next examined *IL16* expression in MyLa cells in co-culture experiments. After co-culture with normal fibroblasts, a significant suppression in *IL16* expression was observed (*p* < 0.03, Fig. 5b). In contrast, co-culture with MF tumor-derived fibroblasts significantly increased *IL16* expression in MyLa cells (*p* < 0.01, Fig. 5b). Several cytokines have been reported to support tumor growth in MF such as IL-13 [51], IL-15 [52] and IL-4 [3, 6]. We next assessed their gene expression in MyLa cells after co-culture with normal and MF fibroblasts. We did not find any significant change in the expression of *IL13* and *IL15* (data not shown). However, whereas *IL4* expression was not significantly altered in MyLa cells after co-culture with normal fibroblasts, co-culture with MF tumor-derived fibroblasts significantly increased *IL4* expression in MyLa cells (*p* < 0.02, Fig. 5c). Furthermore, we assessed the effect of co-culture on the proliferation marker *MKI67*. Interestingly, we observed reduced *MKI67* expression in MyLa cells after co-culture with both MF and normal fibroblasts. This effect was only significant for MF fibroblasts (*p* < 0.01), despite the suppression observed with normal fibroblasts (Fig. 5d).

## Discussion

Studies have shown that tumor growth is preceded or accompanied by activation of local host stroma [53], which plays a major role in disease evolution and response to therapy [54]. CAFs are stromal cells that are abundant in a variety of cancers and have diverse tumor-restraining/promoting roles [55–58]. The cutaneous TME in CTCL includes abundant stromal fibroblast, but their influence on malignant T cell is poorly characterized.

The majority of MF patients experience indolent disease limited to the skin and have an excellent prognosis unless lesions thicken and progress to tumor stage [59]. In contrast, SS is a CTCL with more aggressive and rapidly progressive disease. Early MF skin lesions are characterized by a low burden of malignant T cells accompanied by a reactive immune cell infiltrate. In addition to the immune cells, the skin ME background consists of mesenchymal stromal cells, many of which are fibroblasts [60]. Malignant T cell burden increases with progression from patch to plaque to tumor lesions in MF. Concomitant changes in the TME can be detected, such as increased angiogenesis and stromal fibroblasts expressing matrix metalloproteinase-2 (MMP2) [61]. As disease stage progresses, the skin architecture is disrupted by TME-related changes including epidermal fibrosis, increased Th2 cells and cytokines, and declining expression of Th1 factors [3, 62–64]. Th2-like malignant T cells proliferate in the TME in the presence of MF fibroblasts. Upregulation of CTLA-4 on the surface of malignant T cells further suppresses host immunity [65]. Highlighting the importance of this immune shift in disease progression is that restoring cytokines seen in early MF by treating advanced CTCL with IFN-α and IFN-γ is an effective strategy for treatment [66]. These observations suggest that fibroblasts in early skin lesions may contribute to the indolent nature of early MF by ameliorating disease-promoting gene expression in malignant T cells.

The current study is a first step to elucidate the regulatory role of fibroblasts in CTCL compared to normal fibroblasts. The present study focuses on the dermal fibroblasts between CTCL and normal, and specifically directed at major differences in their interaction with malignant T cells. In the development of CTCL lesions in the skin, there are no privilege sites where lesions have not been observed and in this initial report, we are focused on analyzing properties in fibroblasts that is independent of location. However future studies comparing properties of fibroblasts from different sites in the skin will be important in yielding understanding into the mechanism of progression and site predilection.

In the present study, we isolated fibroblasts from patient tissue, using established methods to elute and analyze fibroblast properties [67–70]. Here we described a novel 2-dimensional co-culture system by incubating primary fibroblasts with malignant T cells. These fibroblasts were rested in culture and were not subjected to any mechanical stress at the time of analysis. In our experiments, both normal and CTCL fibroblast were co-cultured with MyLa cells simultaneously to study disparate properties of these different fibroblast populations.

It has been observed that CAFs greatly influence the TME via the secretion of cytokines and chemokines [71, 72], regulate the plasticity of cancer stem cells [73], and play a significant role in the development of drug resistance [74]. Our findings demonstrated that MF fibroblasts have upregulated expression of *IL32* and *FAPα* compared to normal fibroblasts (Fig. 2a). IL-32 expression has been described in keratinocytes (KCs) in lesional skin of MF patches and plaques, and in atypical T cells in the dermis in MF tumors [36]. IL-32 expression was also detected in KCs in atopic dermatitis [75]. However this study is the first to show differential *IL32* expression in MF fibroblasts compared to normal fibroblasts, which resembles a recent study where IL-32 was found to be abundantly expressed in CAFs in the breast cancer TME [76]. We also showed that fibroblasts in MF differ from normal fibroblasts in the expression of FAP-α, a known CAF marker. We observe expression in MF fibroblasts that is consistent with the previous report of FAP-α in MF lesions [34]. Furthermore, the current study shows increasing expression of FAP-α in more advanced MF stages, and demonstrates for the first time that FAP-α expression can be correlated to stage (Fig. 2f-i) compared to normal donors (Fig. 2b-c).

CTCL presents with a variable clinical presentation, nevertheless there are recurrent morphologic features that can serve as clues in diagnosis and classification. We believe that the distinctive phenotype may reflect signature genes important in Sezary syndrome (SS). Indeed, from transcriptome studies of SS, a number of genes can be frequently detected to be abnormally expressed that are biomarkers [15, 77]. Therefore, we selected a number of biomarker genes to identify CTCL cell lines that are representative of SS for in vitro studies. Of the four cell lines tested (Fig. 3d), only MyLa cells expressed TWIST1, TOX and cytokine genes that are seen in primary SS cells. Based on these results, MyLa cells were chosen as a model SS cells for our experiments.

Using the novel co-culture system in this report, we describe for the first time that normal fibroblasts in co-culture can induce gene expression changes in MyLa T cells, suggesting that fibroblasts from normal skin may suppress disease-promoting gene expression in malignant T cells. Specifically, we showed that fibroblasts can modulate MyLa cells to suppress Th2 genes (Fig. 5a) and enhance Th1-related gene expression (Fig. 4d), suppress expression of the MF biomarkers *TWIST1* (Fig. 4a) and *TOX* genes (Fig. 4b), and inhibit proliferation of MyLa cells (Fig. 5d). The results suggest that fibroblasts in early MF skin lesions may be a microenvironment that is less hospitable for proliferation. We also demonstrated that MF tumor-derived fibroblasts differ from normal fibroblasts in their ability to alter disease-associated gene expression in MyLa cells (Figs. 4a-b and 5a). In contrast to normal fiborblasts, we showed that MF fibroblasts promote expression of Th2 cytokine genes (Fig. 5b-c) and lack the ability to suppress expression of *TWIST1* and *TOX* genes (Fig. 4a-b). Whether this is similar to CAF from solid tumors is unclear. *ACTA2*, which is highly expressed in carcinomas [37–39], is not upregulated in MF fibroblasts compared to their normal counterparts (Fig. 3b). Collectively, these results suggest that signals from normal fibroblasts may mimic the TME of early MF skin lesions, creating an environment inhospitable for proliferation.

Interestingly, these findings are reminiscent of studies on diffuse large B-cell lymphoma (DLBCL), where the finding of stromal gene signature representing fibroblasts and extracellular matrix components associated with good survival, and creating a TME not conducive for lymphoma progression [19]. Lenz et al. identified two sets of stromal gene signatures in DLBCL patients [78]. The stromal-1 gene signature was found to be associated with better survival in DLBCL patients, which includes the genes that are associated with poor survival in other carcinomas [66]. However, the mechanism behind the suppressive effect of normal fibroblasts in CTCL is unclear, and will need further study. Our findings have implications for the understanding of tumor progression in MF in the early stages when malignant cells are sparse, when the skin architecture is preserved with the presence of normal fibroblasts, and when the immune infiltrate consists primarily of nonmalignant (reactive) Th1 cells and cytotoxic CD8+ T cells [3, 79]. The results we found from co-culturing normal fibroblasts with MyLa cells suggest that the Th1 immunophenotype of early stage MF skin lesions may be affected by fibroblasts that increase *IFNG* and *TBX21* expression in T cells. Our observations indicate that MF progression is accompanied by changes in fibroblast phenotype in the TME. We show that MF fibroblasts have a modulatory effect on malignant T cells, supporting their expression of disease-associated cytokines (Fig. 5b-c) and CTCL biomarkers (Fig. 4a-b). When fibroblasts from tumor stage lesions were co-culture with MyLa cells, *IL4* and *IL16* expression (Fig. 5b-c) was increased, which is consistent with a malignant CTCL phenotype [3, 80]. In that setting, high levels of *TWIST1* and *TOX* expression were maintained, in marked contrast to the significant decreases observed when MyLa cells were co-culture with normal fibroblast (Fig. 4a-b). The results from these experiments demonstrate for the first time that fibroblasts from normal skin and MF tumors differ functionally in their ability to modulate gene expression of malignant T cells.

## Conclusions

**Fig. 6:**
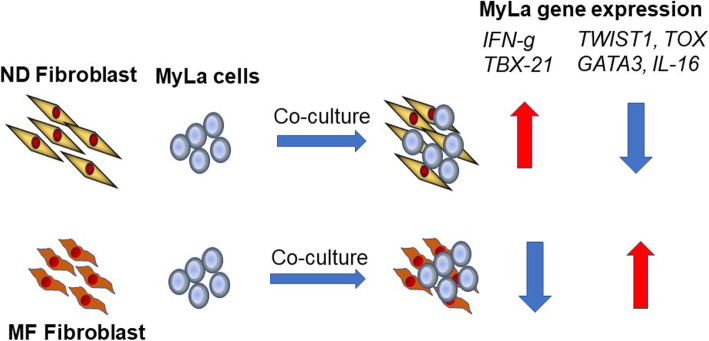
Schematic summary of gene expression changes induced by co-culture of normal and MF fibroblasts with MyLa cells

In summary, our results describe novel activities of normal and MF fibroblasts to modulate gene expression in malignant T cells (Fig. 6). In this report, we focus on the transcription regulation induced by the interaction between fibroblasts and MyLa cells, and not on the function of these gene products such as *GATA3* and *TBX21* that are biomarkers. These novel findings suggest that the TME changes during the evolution of MF tumors, and suggest that fibroblasts in the TME play a role in disease pathogenesis and progression. Whether MF tumor fibroblasts may protect malignant T cells from cytotoxic and genotoxic therapies remains unclear. Further detailed global transcriptomic studies and functional studies such as gene silencing and blocking receptors will be needed to explore the complex interacting pathways between CTCL fibroblasts and primary CTCL cells that alters the regulation of gene expression in CTCL cells. Importantly, understanding of the mechanism important in dysregulated gene expression by skin fibroblasts may lead to the development of new strategies to discover novel compounds for the treatment of CTCL.

## Supplementary Information

**Additional file 1.**

## Data Availability

The datasets used and/or analyzed during the current study are available from the corresponding authors on reasonable request.

## Abbreviations

CTCL: Cutaneous T-cell lymphoma
MF: Mycosis fungoides
SS: Sézary syndrome
TME: Tumor microenvironment
ECM: Extracellular matrix
*VIM*: Vimentin gene
*ACTA2*: Alpha-smooth muscle actin gene
*HSP47*: Heat-shock protein 47 gene
CAFs: Cancer-associated fibroblasts
SA-βGal: Senescence-associated β-galactosidase
ME: Microenvironment
DLBCL: Diffuse large B-cell lymphoma
