# Supplementary figures and images for: Normal and cancer fibroblasts differentially regulate TWIST1, TOX and cytokine gene expression in cutaneous T-cell lymphoma
Source: BMC Cancer. 2021 May 3;21:492. doi: 10.1186/s12885-021-08142-7 (PMC8091512; doi:10.1186/s12885-021-08142-7)

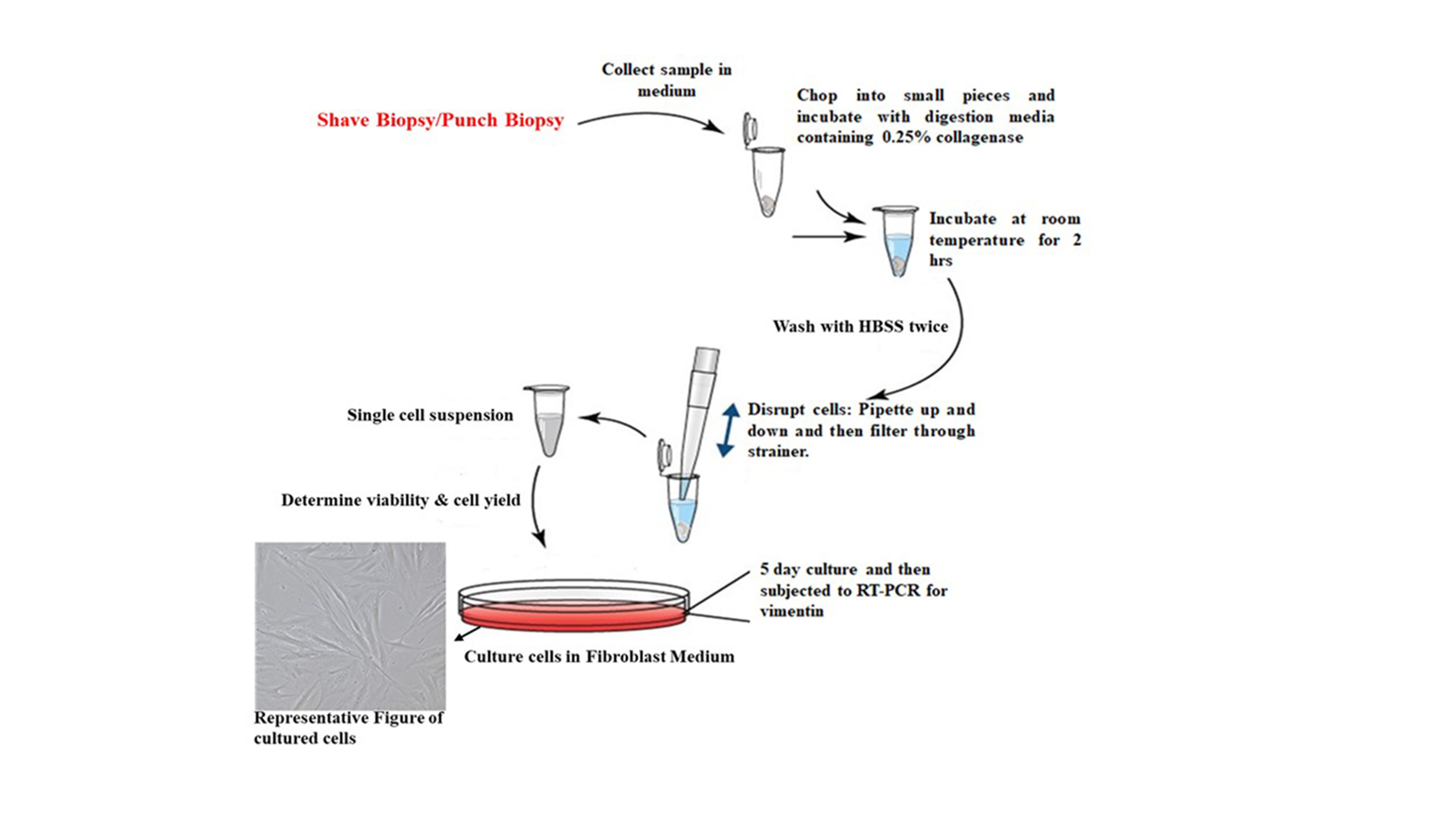

Supplement: Supplementary file 1 — Additional file 1. [file 12885_2021_8142_MOESM1_ESM.jpg]
